# Cancer from the perspective of stem cells and misappropriated tissue regeneration mechanisms

**DOI:** 10.1038/s41375-018-0294-7

**Published:** 2018-10-30

**Authors:** Mariusz Z. Ratajczak, Kamila Bujko, Aaron Mack, Magda Kucia, Janina Ratajczak

**Affiliations:** 1 Stem Cell Institute, Division of Hematology and Oncology, James Graham Brown Cancer Center, University Louisville, 500 South Floyd Street, Louisville, 40202 Kentucky USA; 2 Department of Regenerative Medicine, Center for Preclinical Research and Technology, Warsaw Medical University, Warsaw, Poland

## Abstract

Tumorigenesis can be considered as pathologically misappropriated tissue regeneration. In this review we will address some unresolved issues that support this concept. First, we will address the issue of the identity of cancer-initiating cells and the presence of cancer stem cells in growing tumors. We will also ask are there rare and distinct populations of cancer stem cells in established tumor cell lines, or are all of the cells cancer stem cells? Second, the most important clinical problem with cancer is its metastasis, and here a challenging question arises: by employing radio-chemotherapy for tumor treatment, do we unintentionally create a prometastatic microenvironment in collateral organs? Specifically, many factors upregulated in response to radio-chemotherapy-induced injury may attract highly migratory cancer cells that survived initial treatment. Third, what is the contribution of normal circulating stem cells to the growing malignancy? Do circulating normal stem cells recognize a tumor as a hypoxia-damaged tissue that needs vascular and stromal support and thereby contribute to tumor expansion? Fourth, is it reasonable to inhibit only one prometastatic ligand–receptor axis when cancer stem cells express several receptors for several chemotactic factors that may compensate for inhibition of the targeted receptor? Fifth, since most aggressive cancer cells mimic early-development stem cells, which properties of embryonic stem cells are retained in cancer cells? Would it be reasonable to inhibit cancer cell signaling pathways involved in the migration and proliferation of embryonic stem cells? We will also briefly address some new players in cancerogenesis, including extracellular microvesicles, bioactive phospholipids, and extracellular nucleotides.

## Introduction

Cancer is a serious clinical challenge, and, despite significant efforts to solve this problem, we are unfortunately far from developing cures for the most aggressive solid malignancies and refractory leukemia. We still need to learn more about cancer pathogenesis and find viable targets for therapeutic approaches. Our knowledge about its pathogenesis is mostly derived from gene expression studies, including mRNA, miRNA, protein, and DNA analysis, in clinical specimens harvested from cancer patients [1–4]. Some important information has also been derived from metabolomics studies [5]. However, most of the experimental work in in vitro and in vivo models has been performed in established cancer cell lines. Because it is so difficult to efficiently expand cells isolated from primary growing solid tumors and leukemia, we must rely on this surrogate approach [6].

There are two old sayings that were proposed to understand the pathogenesis of cancer and its progression that are still somewhat apt today. The first is related to cancer progression—that cancer is a “wound that never heals” [7, 8]—and the second, that cancer metastasis embodies the concept of “seed and soil” [9].

The first concept refers to the fact that cancerogenesis and tissue regeneration are somewhat related processes and involve similar mechanisms, including (i) stem cell migration and recruitment and (ii) the activity of chemotactic factors promoting cell motility [7, 8]. In support of this notion, cancer often originates in response to tissue/organ injury or chronic tissue inflammation, and evidence indicates the involvement of misappropriated homeostatic mechanisms that govern normal tissue repair processes and stem cell renewal [10].

By contrast, the “seed and soil” concept addresses the pro-migratory properties of cancer cells and their preferred pattern of metastasis to certain anatomical locations. The migratory potential of cancer cells mimics the mechanisms involved in migration of normal stem cells. Therefore, cancer cells respond to similar stimulating factors as do normal stem cells, follow gradients of similar chemoattractants, and express a similar repertoire of adhesion molecules [9].

Furthermore, the unlimited proliferation potential of cancer-initiating cells mimics embryonic stem cells, with the major difference that malignant cells have defective differentiation potential [11–13]. In past years several factors that stimulate proliferation and metastasis of cancer cells have been identified, and in vivo models have been developed to study metastatic behavior in immunodeficient animals. Nevertheless, despite progress in molecular analysis and extensive in vitro and in vivo studies, there remain many basic questions regarding the biology of growing solid tumors and expanding leukemia, and progress in these areas is necessary for developing more efficient treatment strategies. The most urgent questions concern (i) the cells of origin for developing malignancies, (ii) cancer resistance to therapy and metastasis, (iii) the supportive role of normal stem cells in developing tumors, (iv) the fact that most primitive cancers mimic many features of embryonic cells, and (v) the emerging involvement of new players in cancerogenesis, including extracellular microvesicles (ExMVs), complement cascade cleavage fragments, bioactive phospholipids, and extracellular nucleotides [14–18]. All these questions reflect the need to better understand the complexity of malignant transformation and tumor progression.

Here we will try to address these questions and look at the growing cancer as a misdirected and pathological regenerative process [10, 19]. Some of the most important similarities between tissue regeneration of tumorigenesis are listed in Table 1. In this review, however, we will refrain from discussing the roles of oncogenes and anti-oncogenes in cancer progression, as there have been several excellent reviews published on these issues [1–4].

**Table 1:** Similarities between regeneration and tumorigenesis

Regeneration	Tumorigenesis
Involves normal stem cells	Involves cancer stem cells
Migration of stem cells	Metastasis of cancer cells
Response to similar peptide-based and non-peptide chemoattractants	Response to similar peptide-based and non-peptide chemoattractants
Expression of similar adhesion molecules	Expression of similar adhesion molecules
Recruitment of circulating stem cells	Recruitment of circulating stem cells
Resistance to radio/chemotherapy of normal stem cells	Resistance to radio/chemotherapy of cancer cells
High telomerase activity in stem cells	High telomerase activity in cancer cells

## Cancer as a stem cell disorder?

**Fig. 1:**
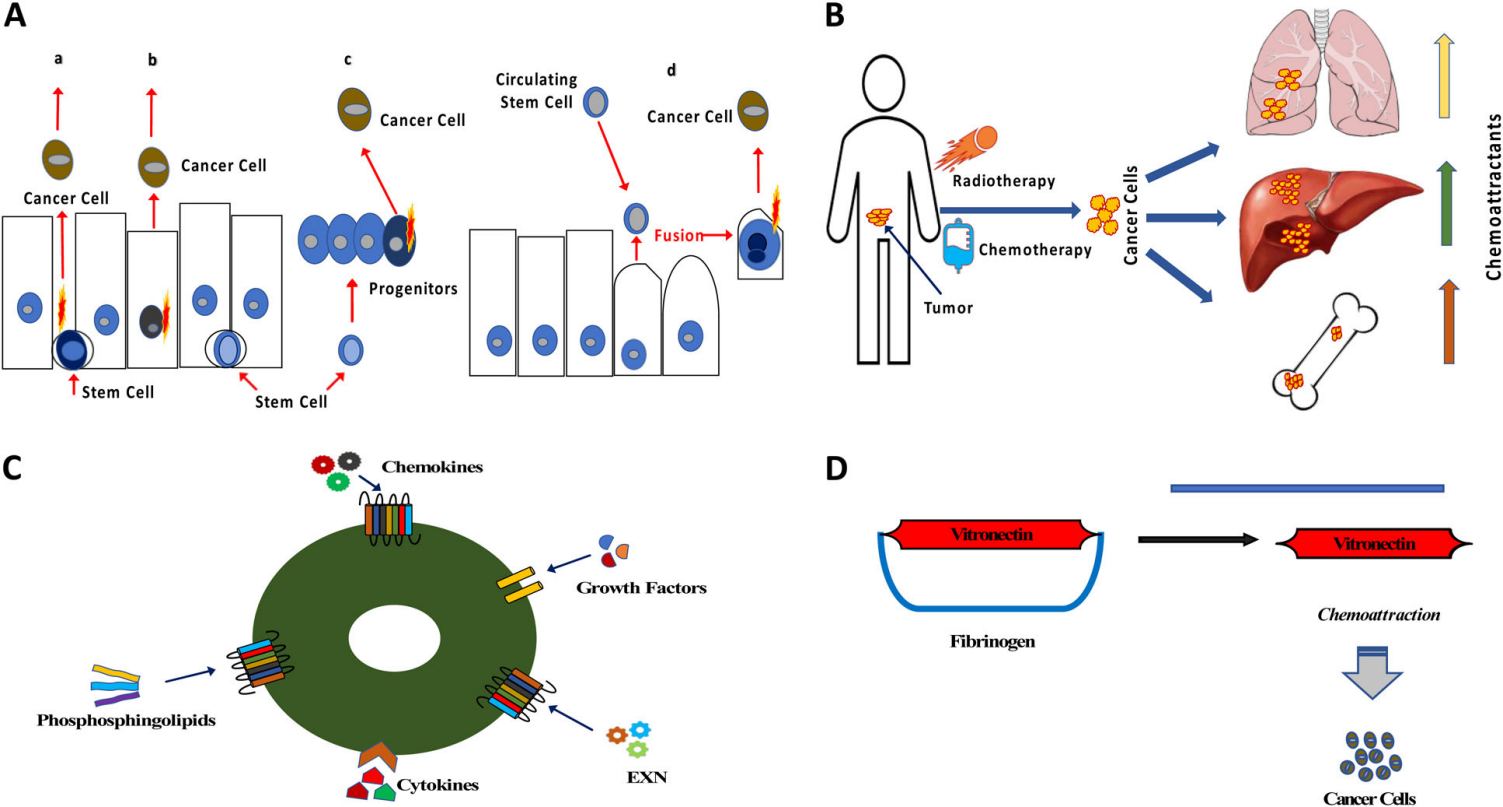
Some new and provocative concepts about tumorigenesis. **a** Potential cancer-initiating cells in solid tumor. As shown, cancer may originate from normal stem cells that have accumulated mutations over time or experienced a potent activating mutation (**a**), from normal somatic cells that have experienced a potent activating mutation (**b**), from mutated progenitor cells (**c**), or as a result of fusion between a circulating stem cell (e.g., a VSEL) and a somatic cell (**d**). **b** Radio-chemotherapy as the triggering mechanism for a prometastatic environment. One of the unwanted side effects of radio-chemotherapy is induction of prometastatic niches in collateral organs. Therapy-resistant cells may respond to chemoattractants upregulated in prometastatic niches and metastasize to such areas. **c** Cancer cells express functional receptors for several different chemoattractants. Since cancer cells respond to chemotactic gradients of several chemoattractants, it is problematic to target one receptor–ligand metastatic axis only. The ideal antimetastatic therapy should target common mechanisms downstream of chemotactic receptors. **d** Vitronectin is a potent chemoattractant of tumor cells to lymphatics and body cavities. Vitronectin is not only an adhesion molecule but also a potent chemoattractant that is bound by fibrinogen. Cancer cells respond robustly to vitronectin and metastasize preferentially to lymphatics and body cavities where the concentration of fibrinogen is low, and vitronectin is thereby released from its inhibitory complex with fibrinogen

The identity of the cells that give rise to malignancies is still disputed in the literature [20, 21]. It has been proposed that cancer may be initiated by (i) normal tissue-residing stem cells, (ii) an expanded proliferating population of progenitor cells, (iii) normal adult cells that have acquired mutations conferring them with an immortal phenotype, (iv) heterokaryons produced by the fusion of normal circulating stem cells with somatic cells, and (v) rare embryonic remnant cells residing in the body (Fig. 1a). However, it is important to note that there is no common mechanism or cell type that acquires a malignant phenotype and the mechanism for initiation of malignancy differs from tumor to tumor.

The concept that normal stem cells potentially give rise to cancer is based on the assumption that some period of time is needed to accumulate mutations at the single-cell level until they reach a critical threshold that endows the cell with a malignant phenotype [20, 21]. The time requirement for this to occur suggests that the affected cells are within the population of stem cells residing in adult tissues. Based on this possibility, malignancy may originate in the compartment of normal, long-living stem cells and result from their maturation arrest and uncontrolled proliferation [20]. These normal stem cells residing in a given organ including bone marrow could accumulate consecutive mutations and pass them to their daughter stem cells, which at a certain point descend into uncontrolled proliferation and become tumor-initiating stem cells. By contrast, differentiated somatic cells usually have a short half-life, as seen for example in gastrointestinal epithelium, epidermis, or the mature cells in hemato/lymphopoietic system and are eliminated relatively rapidly from the body.

Nevertheless, it is still problematic to identify cancer stem cells in growing malignancies with 100% certainty and even more difficult to demonstrate their in vivo tumor-initiating properties. Until now, positive results in testing for tumor-initiating potential in animal models have been obtained by implanting clumps of cells or cell suspensions  isolated from growing tumors [22]. It is possible that the cancer-initiating stem cells present among the implanted cells need crosstalk with other cancer cells to establish tumors in experimental animals or that current animal models to study human tumor cells are still inadequate for assaying tumor-initiating potential at the single-cell level.

Cancer-initiating cells may also originate in compartments of expanded proliferating progenitor cells that, if accumulated in the tissues, could be an easy target for cancerogenic events [20]. This model has been proposed for some hematopoietic malignancies [21]. It is also possible that some cancer-initiating cells originate directly from mutated somatic cells that, due to a key mutation, acquire an immortal phenotype [20, 21].

Another interesting concept has been proposed recently for the origin of aneuploid cancers [23]. Specifically, it has been postulated that cancer originates after fusion of two cells that form a heterokaryon, a cell carrying a duplicate set of chromosomes. Such cells usually die out, although it is possible that, after excluding the additional chromosomes, they reach a state of aneuploidy that supports proliferation and an immortal state driven by the remaining set of chromosomes, supported in some cases by chromosomal rearrangements [23]. This concept also has an important implication for the possibility that such cell fusion could be the result of joining a normal somatic cell with a circulating stem cell [24]. For example, it has been proposed that gastric cancer due to chronic infection by *Helicobacter pylori* originates from circulating stem cells that are seeded into the inflammatory microenvironment of the stomach mucosa [24]. It would be interesting to see whether such a fusion process in tissues affected by local inflammation could be initiated by fusion between a circulating stem cell and a somatic cell residing in a given organ.

Finally, it is also possible that rare malignancies originate from embryonic remnants that survived embryogenesis [13], and this possibility will be discussed later in this review in light of the presence of very small embryonic-like stem cells (VSELs) residing in adult tissues [13].

## Do established cancer cell lines contain a distinct population of rare cancer stem cells?

The concept that stem-like cells exist within primary tumor tissues has stimulated a search to identify candidate cells in established cancer cell lines [24, 25]. Therefore, since a majority of experimental work with cancer cells has been performed on established cancer cell lines, one of the most important questions is whether cancer cell lines contain a distinct subpopulation of primitive cancer stem cells that maintain their expansion in in vitro cultures [24, 25]. In fact, it has been reported in the literature that cells in growing cancer cell lines may express markers, such as CD133 and CXCR4, that are highly expressed in normal hematopoietic and non-hematopoietic stem cells.

To address this issue, we performed studies on the established human ovarian cancer cell line A2780 and the embryonal carcinoma cell line NTera2 and evaluated the potential stemness of cells sorted according to expression of the cell-surface stem cell markers CD24 plus CD44 and CD133 plus SSEA4, respectively [24, 25]. It has been postulated that while human ovarian cancer stem cells are CD24^+^CD44^+^, human embryonal carcinoma stem cells are CD133^+^SSEA4^+^. To evaluate the stem cell potential of these cells, we sorted these cells according to four phenotypes: CD24^–^CD44^–^, CD24^+^CD44^–^, CD24^–^CD44^+^, and CD24^+^CD44^+^ (A2780 cells) and CD133^−^SSEA4^–^, CD133^+^SSEA4^–^, CD133^–^SSEA4^+^, and CD133^+^SSEA4^+^ (NTera2 cells) and observed in in vitro and in vivo assays different properties of cells expressing both, one, or neither of these antigens [24, 25]. We found that sorted cells enriched for stem cell markers possessed higher migratory and adhesive properties than cells negative for these markers [24, 25]. This result supported the functional properties thought to be characteristic of metastatic cancer stem cells [26].

However, when we sorted these cells as single cells according to their respective phenotypes including cells that did not expressed putative cancer stem cell markers and expanded them ex vivo under limiting dilution conditions, we found that all gave rise to clones that were comparable to the parental cell lines in terms of surface antigen expression, and, importantly, all four phenotypes were again reestablished in the expanded clones [24, 25].

Therefore, our results suggest that, within established cancer cell lines, the phenotypes of cells with cancer stem cell markers are not fixed and instead fluctuate during cell line expansion [24, 25]. This is an important observation relevant to experiments performed with “putative solid cancer and leukemia stem cells” purified from established cancer cell lines, as these properties of “putative cancer stem cells” change with the cell cycle and fluctuate during expansion in vitro or after inoculation into animals. Based on this finding, we should consider every cell in established cell lines as a stem cell that, in most cases, can re-establish a clone resembling the parental cell line [24, 25].

## Does radio-chemotherapy unintentionally induce a prometastatic environment?

The leading cause of death from cancer is tumor expansion, which usually leads to metastasis of malignant cells and tumor dissemination to the vital organs and may cause organ failure or cachexia [26]. It is well-known that cancer cells, like normal stem cells, employ several mechanisms that render them resistant to radio-chemotherapy [27]. The most important are efficient DNA repair mechanisms, expression of multidrug resistance genes (ABC transporters), and expression of enzymes such as aldehyde dehydrogenase (ALDH) that metabolize cytostatic drugs [27, 28]. These radio-chemotherapy-resistant cells are “seeds” in the “seed and soil” concept of cancer metastasis [9].

In this context, accumulating evidence suggests that growing tumors contain rare, primitive cells (related to cancer stem cells?) that are highly mobile and, if they survive radio/chemotherapy, are responsible for tumor regrowth and form distant metastases after treatment [20, 21]. What is also very important, these cancer cells, like normal stem cells, respond by chemotaxis to several chemoattractants circulating in peripheral blood or lymph and are upregulated in pre-metastatic niches in remote anatomical locations [16, 29, 30]. This chemotactic microenvironment, which creates a fertile “soil” for cancer metastasizing cells in different organs, may be induced as an unintentional side effect in response to radio-chemotherapy [29, 30].

This phenomenon has been well recognized in bone marrow transplants, in which myeloablation of existing hematopoiesis is performed by irradiation before transplantation of hematopoietic stem cells or administration of busulphan or cyclophosphamide and  upregulates several stem cell chemoattractants in the bone marrow microenvironment [29]. These factors include stromal-derived factor 1 (SDF-1), hepatocyte growth factor/scatter factor (HGF/SF), vascular endothelial growth factor (VEGF), and bioactive lipids such as sphingosine-1-phosphate (S1P) and ceramide-1-phosphate (C1P) [29]. They chemoattract normal stem cells after hematopoietic transplant, but if upregulated after radio/chemotherapy they are also potent chemotractants for cancer cells. For example, as demonstrated in several studies, SDF-1 chemoattracts CXCR4^+^ cancer cells to bone, which leads to initiation of bone metastases [29, 30].

We predicted that a similar response would accompany the toxic effects of radio-chemotherapy in other organs sensitive to damage, including lung or liver (Fig. 1b). Surprisingly, the possibility of induction by these therapies of the expression of several factors in various organs that together create a prometastatic microenvironment was not widely acknowledged. In our studies performed with the human ovarian cancer cell line A2780, we found that total body irradiation or administration of cisplatin increases the metastatic spread of human ovarian cancer cells transplanted into immunodeficient mice compared with control animals unexposed to irradiation or cisplatin [29, 30]. This spread was accompanied by an increase in several chemoattractants in peripheral tissues. Interestingly, we were able to decrease this metastatic spread by employing anti-inflammatory treatment with non-steroid (ibuprofen) or steroid-based (prednisone) anti-inflammatory drugs at the time of radio-chemotherapy administration [29, 30]. Recently we demonstrated that a similar effect occurs in vivo after radio-chemotherapy in an experimental model of lung cancer [31].

Based on this finding, we propose that a radio-chemotherapy-induced prometastatic microenvironment plays an important role in the metastasis of cancer cells that are resistant to treatment and have characteristics of highly migratory cancer stem cells. By inducing a prometastatic microenvironment and prometastatic niches, radio- or chemotherapy treatment could be a double-edged sword that limits the therapeutic benefits of anti-cancer treatment. However, this disturbing concept needs further study.

## Do normal circulating stem cells support tumor progression?

As mentioned above, mutated normal stem cells may be the origin of certain malignancies. In addition, they may also contribute indirectly to tumor progression in other ways. It is well-known that the growing solid tumor is constantly remodeling tissue due to the hypermetabolism of cancer cells and overall hypoxic conditions. Its expansion and growth depend on two important processes: (i) proper vascularization and (ii) formation of supportive stroma. However, the origin of cells involved in the development of new vessels and stroma in tumors is somewhat unclear. They may be recruited from the tumor by neighboring tissues or originate de novo from circulating stem cells recruited to the tumor tissue [13].

Drawing on the analogy of a growing tumor as a wound that never heals [7, 8], one can imagine that tumor tissue is sensed by normal stem cells as a damaged organ. It is well-known that during organ/tissue injury and inflammation stem cells are released from bone marrow as well as from other organs into circulation and may play a role in ameliorating tissue damage [32]. This phenomenon was previously demonstrated in heart infarct, liver damage, and stroke [33]. Besides hematopoietic stem cells (HSCs), mesenchymal stromal cells (MSCs), endothelial progenitor cells (EPCs), and VSELs are released in such clinical situations. All these stem cells may mistake the growing tumor for damaged tissue and generate cells that provide new vessels and stroma [13]. On the other hand, circulating stem cells may secrete soluble factors and release ExMVs, which promote proliferation, survival, and an increase in the metastatic potential of cancer cells [14].

These unwanted tumor progression-promoting effects of normal circulating stem cells provide support for the concept that mechanisms operating during normal regeneration may be misappropriated to occur in pathological situations during cancerogenesis [10, 19].

## Is there a rationale for strategies that inhibit only one receptor–chemottractant axis?

The major problem with cancer progression is the inherent ability of cancer cells to migrate and establish distant metastases, which is responsible for >90% of cancer-associated mortality [34]. As mentioned above, this ability to metastasize correlates with the presence in a growing tumor of cancer cells with a more malignant phenotype that express certain markers of normal stem cells [20, 21, 29]. Overall, this process in many respects mimics the migration of normal stem cells during organogenesis in the developing embryo. Therefore, one of the most important clinical problems is to limit the metastatic potential of cancer cells. Several factors, including cell migration-promoting cytokines, chemokines, growth factors, bioactive lipids, extracellular nucleotides, and even H^+^ ions, were found to influence the metastasis of solid cancer and leukemia cells [29, 34]. These prometastatic factors activate the corresponding receptors, including cytokine receptors, tyrosine kinase receptors, and G protein-coupled receptors. In response to activation of these receptors, similar signaling pathways are initiated that are involved in the regulation of cell migration and adhesion.

This plethora of potential pro-migratory chemotactic factor–receptor axes demonstrates the existence of significant redundancy in the chemoattractants for cancer cells (Fig. 1c). In spite of this, significant effort has been made to demonstrate in in vitro and in vivo animal models the crucial role of specific prometastatic factor–receptor axes in metastasis. Moreover, based on this data, new drugs targeting one receptor or one chemoattractant have often been developed.

For several years our team has been interested in the role of different chemoattractant–receptor axes in a model of metastasis for human rhabdomyosarcoma (RMS) tumor that frequently infiltrates bone marrow [34]. We evaluated the efficacy of different peptide-based chemoattractants, bioactive phospholipids, and extracellular nucleotides, and came to the conclusion that targeting a single receptor–ligand prometastatic axis will not effectively prevent metastasis of RMS, and that we should seek other more effective therapeutic options based on targeting common signaling pathways downstream of these receptors. We propose that results obtained with our RMS cell metastasis model are also relevant to other types of malignancies, as significant redundancy in prometastatic ligand–receptor axes exists for almost all tumor types studied so far [34].

Therefore, redundancy in the responsiveness of cancer cells to various chemoattractants provides a serious challenge to the rationale for inhibiting single receptor–chemoattractant axes (Fig. 1c).

## How much embryonic stem cell potential is present in cancer cells?

Highly malignant and metastatic cancers express several markers characteristic of early-development stem cells. These markers include expression of embryonic transcription factors such as Oct-4, secretion of human chorionic gonadotropin (hCG) and carcinoembryonic antigen (CEA), and expression of cancer testis antigens (CTAs) [12, 13]. This expression may indicate that some tumors originate in cells related to embryonic cells that are present in postnatal tissues [13].

In the 19th century, Rudolf Virchow and Julius Cohnheim proposed the “embryonic rest” hypothesis of cancer development [35, 36]. These famous pathologists hypothesized that adult tissues contain embryonic remnants that normally lie dormant but can be activated to become malignant. In support of this original concept, other pathologists at the beginning of the 20th century, such as Wright, proposed a germinal cell origin of Willm’s tumor, and John Beard proposed that tumors may arise from activated trophoblasts displaced during embryogenesis or even from germ cells [13].

This 150-year-old hypothesis is somehow supported by the recent discovery of VSELs residing in postnatal tissues [37, 38]. We envision that the VSELs discovered by our team and confirmed recently by other independent groups could somehow reconcile the embryonic rest hypothesis with current theories that cancer is a disorder initiated in some cases by early-development stem cells, as VSELepibla/epiblast cell markers at the mRNA and protein levels [38].

In support of this hypothesis of cancer development, various tumor cell types, including gastric, lung, liver, renal, and bladder carcinomas, pediatric sarcomas, and germinal tumors, frequently express the abovementioned CTAs (~40 identified so far), which are normally expressed in epiblast/germline cells. Thus, the presence of these markers in solid tumors could indicate that cancer originates from rare, early-development epiblast/germline-related cells [13]. Importantly, VSELs express several CTAs (e.g., MageB3, Ssbx2, and BORIS) [13] and express the embryonic transcription factor Oct-4 in the nucleus and stage-specific embryonic antigen 4 (SSEA-4) on the cell surface [38].

Based on these findings and others mentioned earlier in this review, VSELs, which are a highly mobile population of normal stem cells released during stress situations from bone marrow into peripheral blood, could act in neovascularization and stromalization of a growing cancer [13]. We also hypothesize that if VSELs acquire appropriate mutations, they may give rise to cancer stem cells and initiate the growth of teratomas and teratocarcinomas, germinal tumors, or pediatric sarcomas (e.g., rhabdomyosarcoma, neuroblastoma, nephroblastoma) [13, 38]. In addition, circulating VSELs may fuse with somatic cells in tissues that are affected by inflammation and give rise to heterokaryons, which give rise to aneuploid cancer stem cells, as demonstrated in Fig. 1a. However, the direct involvement of VSELs in all these proposed scenarios of cancerogenesis needs more experimental confirmation. It is also an open question if VSELs that show hematopoietic specification [38] may give rise to leukemia.

Moreover, considering the fact that several types of cancer cells express several features of embryonic stem cells, it is not surprising that these cells exhibit dependence on many of the same primitive regulatory pathways operating in embryonic stem cells [11, 13, 39]. The most important embryonic signaling pathways, including Notch, Wnt, and Hedgehog, which regulate normal and malignant stem cell maintenance and growth, could become targets for anti-cancer treatment strategies. This concept has been well presented in another recent review paper [39].

## New players in cancer progression and metastasis

For many years the main focus in solid cancer and leukemia research was devoted to the roles of peptide-based growth factors and chemoattractants, including cytokines and chemokines. It is now clear that in addition to these molecules, an important role is also played by factors from the phospholipid family as well as extracellular nucleotides [40, 41]. All these new players involved in the migration of cancer cells are also involved in the migration of normal stem cells [42].

Additional evidence has accumulated that in the coagulation and complement cascades zymogen proteins and their cleavage products directly or indirectly affect cancer progression and metastasis [43]. Complement cascade cleavage fragments are also known to be involved in solid tumor and leukemia stem cell migration as well as in the development and regeneration of various tissues [44].

Moreover, evidence has accumulated that ExMVs are shed from the cell surface or secreted as exosomes from the endosomal membrane compartment [45, 46]. ExMVs may directly stimulate tumor cells as signaling packets, and since they possess chemotactic activity, chemoattract tumor cells as well [47]. ExMVs may also deliver mRNA, miRNA, proteins, and bioactive lipids to cancer cells, which stimulate proliferation and make these cells more resistant to therapy [48]. Similar ExMVs-mediated mechanisms operating in cancer are also involved in crosstalk between normal cells during organ development and tissue regeneration [48].

We have also recently uncovered the pivotal role of vitronectin in metastasis [49]. The propensity of cancer cells to infiltrate lymphatic and lymph nodes and preferentially migrate to body cavities (e.g., peritoneal or pleural cavity) is still not well explained. An interesting possible explanation for these phenomena could be the action of vitronectin, which for many years was considered to be an important adhesion molecule [49]. Recent work indicates that vitronectin, besides being an adhesion molecule, also has very strong chemotactic activity against several types of tumor cells. What is also interesting, this chemotactic activity of vitronectin is suppressed by binding to fibrinogen (Fig. 1d). This novel observation may explain the preferential metastasis of cancer cells to lymph or body cavities where the concentration of fibrinogen is relatively low, and thus vitronectin is not bound to fibrinogen and as free protein can chemoattract cancer cells [49].

## Conclusions

Mounting evidence suggests that there exists a thin line between regeneration and tumorigenesis. In this review, we presented parallels between normal stem cells and cancer cells as well as common mechanisms that are employed by normal stem cells for their developmental migration or tissue regeneration and are subsequently missapropriated by cancer cells during metastasis. Both normal and cancer cells respond to similar chemoattractants and proliferate in response to similar growth factors. Similar receptors also direct the migration of these cells. Therefore, we can consider cancer as a wound that never heals and envision that normal and cancer stem cells are seeds that migrate through the blood stream and lymphatics looking for fertile soil in which to grow.
